# Evaluation of alpha-synuclein immunohistochemical methods for the detection of Lewy-type synucleinopathy in gastrointestinal biopsies

**DOI:** 10.1186/s40478-016-0305-8

**Published:** 2016-04-04

**Authors:** Anne-Gaëlle Corbillé, Franck Letournel, Jeffrey H. Kordower, John Lee, Elisheva Shanes, Michel Neunlist, David G. Munoz, Pascal Derkinderen, Thomas G. Beach

**Affiliations:** Inserm, U913, Nantes, F-44035 France; Nantes University, Nantes, F-44035 France; Department of Neurology, CHU Nantes, Nantes, F-44093 France; CHU Angers, Neurobiology and Neuropathology Laboratory, Angers, F-49033 France; Université of Angers, UPRES EA3143, Angers, F-49033 France; Department of Center for Brain Repair, Department of Pathology, Rush Medical College, Chicago, IL USA; Department of Pathology, NorthShore Medical Group, Evanston, IL 60201 USA; Laboratory Medicine, St. Michael’s Hospital, University of Toronto & Li Ka Shing Knowledge Institute, Toronto, ON Canada; Banner Sun Health Research Institute, Sun City, AZ USA; Department of Neurology, CHU Nantes, 44093 Nantes, France

**Keywords:** Parkinson’s disease, Gastrointestinal biopsies, Alpha-synuclein, Biomarker

## Abstract

The observation showing that Lewy type synucleinopathy (LTS), the pathological hallmark of Parkinson’s disease (PD), is found in the gut of almost all PD subjects led to a substantial amount of research to develop a diagnostic procedure in living patients based on endoscopically obtained gastrointestinal biopsies. However, the existing studies have provided conflicting results regarding the sensitivity and specificity of gastrointestinal biopsies for the detection of LTS. We therefore undertook a multi-center staining and blinded judging of a common set of slides from colonic biopsies to determine the optimal protocol for the detection of LTS. Four different immunohistochemical methods, developed in four separate expert laboratories, were evaluated for their sensitivity and specificity to detect enteric LTS. Test sets of formalin-fixed, paraffin-embedded sections from biopsies of 9 PD subjects and 3 controls were stained with the 4 methods and graded by 4 different observers. Four types of staining morphology (granular staining in the *lamina propria*, perivascular/vascular wall staining in the submucosa, lacy-granular pattern in the submucosa and epithelial cell nuclear staining) were variably observed in the slides stained by the 4 methods. Positive alpha-synuclein staining was observed by all 5 judges in most of the slides from control cases, regardless of the staining methods that were used. Moreover, none of the tested method or staining pattern had a specificity and sensitivity more than 80 % regarding to PD. Overall, our study suggest that the tested methods are not adequate for the prediction of PD using gastrointestinal biopsies. Future studies are warranted to test new immunostaining methods.

**Electronic supplementary material:**

The online version of this article (doi:10.1186/s40478-016-0305-8) contains supplementary material, which is available to authorized users.

## Introduction

Since the discovery of aggregated and phosphorylated alpha-synuclein as the primary component of Lewy pathology, immunohistochemistry for alpha-synuclein has become the histological technique of choice for the diagnosis of Parkinson’s disease (PD) [1, 2]. Several autopsy studies, showed that the distribution of Lewy-type synucleinopathy (LTS) is much greater than formerly appreciated extending to peripheral autonomic neuronal circuits [2, 3]. The presence of LTS in peripheral neuronal tissues accessible to biopsies provides new opportunities for the development of original biomarkers that will directly assess the pathological process in living patients [4, 5]. Among these peripheral autonomic neuronal circuits, the enteric nervous system displays several specific features that make it a prime candidate tissue for being a histopathological marker of PD as (i) it is an integrative neuronal network whose organization and complexity closely resemble that of the central nervous system (ii) it has been shown to be affected by the pathological process in the vast majority of PD patients [3, 6–9] (iii) it is readily, repeatedly and safely accessible by endoscopic biopsies in living patients [10, 11]. This logically led laboratories to develop specific methods for the detection of LTS in routine gastrointestinal biopsies. However, the diversity of methodology between studies, especially regarding the immunohistochemical methods used, has led to conflicting results and discrepancies regarding the sensitivity and specificity of gastrointestinal biopsies for the detection of LTS [12–17]. The aim of our research project was therefore to test several different immunohistochemical methods for the detection of LTS in colonic biopsy with the objective of identifying a highly sensitive and specific technique that might be widely and readily used in different laboratories. For this purpose, a panel of expert investigators have participated, based on their published work using alpha-synuclein immunohistochemistry [2, 13, 18, 19] and were asked to stain identical sets of paraffin-embedded sections from colonic biopsies with their own optimized method.

## Materials and methods

### Selection of subjects and formalin-fixed paraffin-embedded blocks

Nine PD patients were included in this study, chosen from among 29 subjects that had been recruited from the movement disorder clinic in Nantes University Hospital, France for a previously published study of enteric synucleinopathy in wholemount mucosal-submucosal biopsies [12]. From the same study, a set of 3 subjects (out of 10 in the original study) requiring a total colonoscopy for colorectal cancer screening were included as controls. The clinical characteristics of study subjects are summarized in Table 1.

**Table 1:** Clinical and pathological characteristics of study subjects

Diagnosis	Age Mean (range)	Gender M:F	Disease duration (y) mean (range)	Wholemount synucleinopathy density grade mean (range)	FFPE synucleinopathy density grade mean (range)
Control (*n* = 3)	64 (61–68)	2:1	N/A	0	0
PD (*n* = 9)	66 (47–71)	8:1	12.2 (4–20)	1.6 (0–2)	1.9 (0.5–3)

Presumptive synucleinopathy in the formalin-fixed, paraffin-embedded (FFPE) sections was graded on a 0–3 scale. Wholemount synucleinopathy scores shown are from the original published study [12] and were graded as 0–2 based on the number of positive biopsies out of the 4 that were taken from each subject (the maximum assigned score was 2)

The 3 control and 9 PD subjects were chosen after screening sections from their archived descending colon biopsies stored in formalin-fixed, paraffin-embedded (FFPE) blocks. Screening was performed by AGC and FL of the Neuropathology Laboratory in Angers, France. Tissue blocks were fixed in 10 % (v/v) formalin for 48 h, embedded in paraffin and cut at 4 μm using a Leica RM125 microtome. Sections were collected on slides (Superfrost Plus, VWR, Fontenay sous Bois, France) and stained with an immunoperoxidase method for the detection of phosphorylated alpha-synuclein (Table 2). Global grading of all staining, regardless of morphology, was assigned using a 0–3 semi-quantitative density scale. PD cases were divided into 3 groups with 3 subjects in each group, one group with sparse staining density (PD group 0), one with moderate staining density (PD group 1) and one with high staining density (PD group 2). Control subjects had minimal staining densities (Table 1)

**Table 2:** Immunoperoxidase methods used by the central laboratory (AGC/FL) and the three testing laboratories

	Primary antibody	Epitope exposure	Signal development
AGC/FL (Angers, France)	p-synuclein, #64 WAKO, Osaka 1:30,000	Formic acid (80 %) 20 min	BOND MAX Leica Biosystems, Newcastle Upon Tyne, U.K.
TB (Sun City, AZ)	p-synuclein, polyclonal H. Akiyama, Tokyo 1:10,000	Proteinase K 1:100 37 ° C, 20 min	ABC, DAB Vector Laboratories
JK (Chicago, IL)	Alpha-synuclein, LB509 Invitrogen 1:500	Formic acid (88 %) 20 min	ABC, DAB Vector Laboratories
DM (Toronto, Canada)	p-synuclein, #64 WAKO, Osaka 1:12,000	Ventana Protease 2 72 ° C, 8 min Citrate buffer, pH 6 4 90 ° C, 4 min	Ventana Benchmark Ultra, Optiview multimer and amplifier

p-synuclein = alpha-synuclein phosphorylated at serine 129; ABC = avidin-biotin peroxidase complex; DAB = 3, 3’ di-aminobenzidine

.

None of the control subjects had a history of neurological or psychiatric diseases, none had abnormalities on neurological examination and on telephone interviews done in 2014 none had subsequently developed parkinsonian signs or symptoms. The study protocol was approved by the local Committee on Ethics and Human Research (*Comité de Protection des Personnes Ouest VI*), and registered on ClinicalTrials.gov (identifier NCT00491062). Written informed consent was obtained from each patient and from each normal volunteer.

### Preparation of test slides and distribution to participating labs

**Fig. 1:**
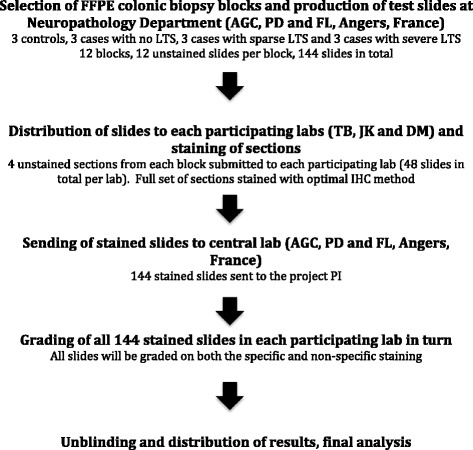
Design of the study

The design of the study is depicted diagrammatically in Fig. 1. Four unstained sections (4 μm) from each block (each block containing one biopsy tissue fragment) were sent by the central laboratory (AGC and PD) along with a brief methods section describing how the tissue was sampled to the 3 other participating testing laboratories (48 slides per lab, 144 slides in all). Each participating lab (TB, DM and JK) including the central laboratory stained the full set of sections with their optimal immunoperoxidase method for enteric synucleinopathy (Table 2). Blocks were assigned a number from 1–12 using a random number generator in order to blind evaluators to diagnosis.

### Slides grading and statistical analysis

**Fig. 2:**
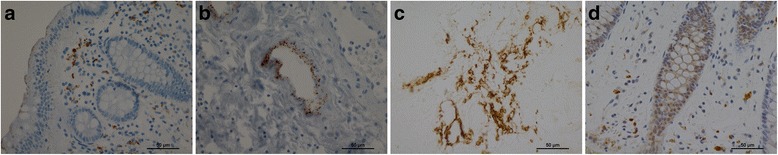
Photomicrographs of LTS immunostaining in colonic biopsies sections, illustrating the 4 staining patterns that were graded. **a** *lamina propria* granular pattern, Template 1; **b** perivascular/vascular pattern, Template 2; **c** lacy-granular pattern, Template 3; **d** epithelial nuclear pattern, Template 4. Scale bar: 50 μM

The stained slide sets were initially examined by TB, who then constructed four different semi-quantitative grading templates (Fig. 2 and Additional file 1: Figures S1 and S2) with associated scoring tables for each of the four major morphological staining types present. The four different staining patterns were: (1) granular staining in the *lamina propria* of the mucosa (2) perivascular/vascular wall staining with a fine dotted pattern, in the submucosa (3) lacy-granular pattern in the submucosa (4) epithelial cell nuclear staining. The photomicrographs in Fig. 2 illustrate each staining pattern. As some of the staining patterns (types 2 and 3) were present only in the submucosa, or depended on the presence of blood vessels in the submucosa, the presence or absence of submucosa or blood vessels was recorded for each biopsy. No assumptions were made as to whether any of the staining patterns seen might represent specific or non-specific staining of pathological alpha-synuclein deposits, and all slides were graded for each pattern by each participating test lab in turn (TB, JK, and DM and one additional judge: John Lee, MD, PhD, of the Department of Pathology, NorthShore University Health System, Evanston, IL). The slides as well as the scoring tables were then returned to PD at the central laboratory. Density scores of none, sparse, moderate and frequent were converted to numerical values (0–4) for statistical analysis. Sum and average score per biopsy were calculated. All labs submitted their grading to the project principal investigator and results were analyzed after unblinding.

Statistical analyses were performed using intraclass correlation coefficient for judge ratings of staining densities, for each laboratory (TB, JK, FL, DM) and staining template pattern (LP = *lamina propria* granular pattern; EC = endothelial cell nuclear pattern; LG = lacy-granular pattern; PV = perivascular-vascular pattern) were determined. The diagnostic accuracy of each staining pattern, in terms of being able to predict a diagnosis of PD, was calculated from each individual judge’s results and for the average of all judge’s results, for all applicable staining patterns seen on slides from each testing laboratory as well as from the central laboratory.

## Results

### LTS staining pattern and density scores

**Fig. 3:**
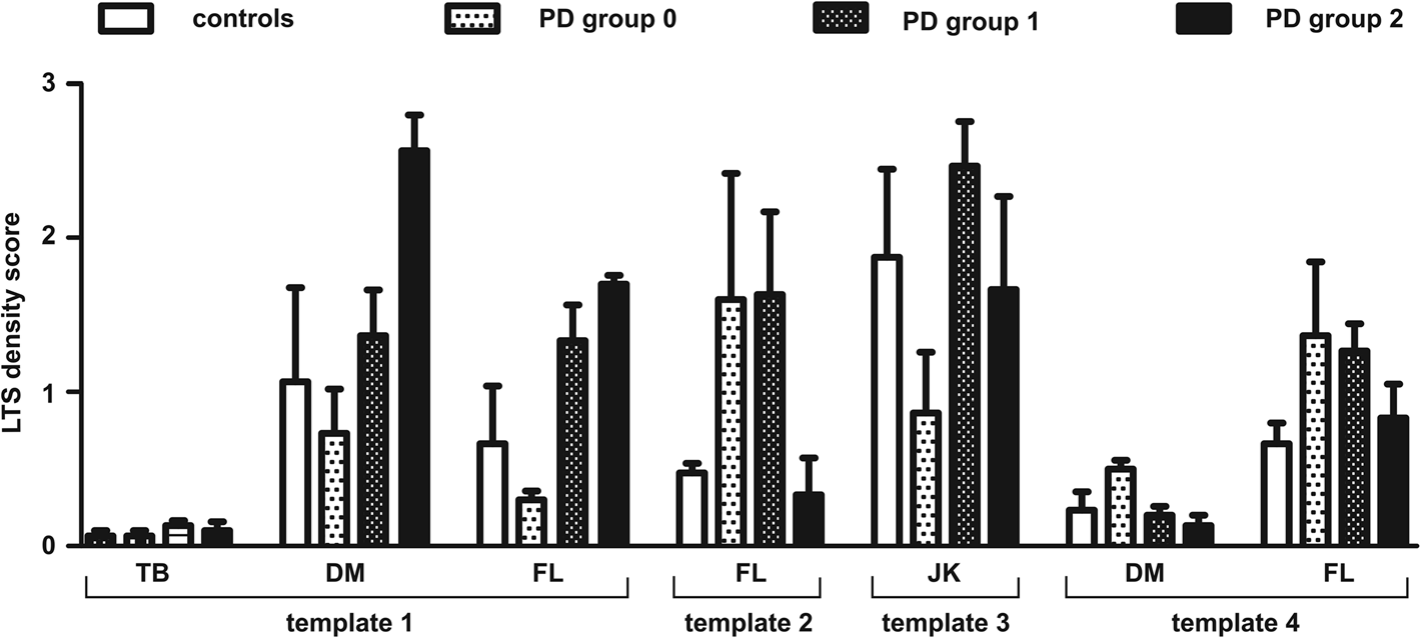
LTS immunostaining scores for each template. Bar graph represents the LTS mean score for each group for each template in all methods. Data correspond to mean ± SEM of the 3 cases per group. PD group 0, 1 and 2 correspond to PD cases with sparse, moderate and high presumed LTS staining. Note that some templates were not observed in some methods. Template 1 : lamina propria granular pattern; Template 2 : perivascular/vascular pattern; Template 3: lacy-granular pattern; Template 4 : epithelial nuclear pattern

The density scores of the 4 raters were averaged to obtain a mean score of LTS staining per biopsy. Figure 3 represents the mean of density score by groups (controls and PD groups) for each staining pattern. Additional file 1: Table S1 shows the results of individual judge scores for the staining done by each test laboratory as well as by the central laboratory, for each scoring template. The four types of staining morphology were variably present in the slides stained by the 4 methods. The granular *lamina propria* staining was seen to some extent in all slide sets. The perivascular-vascular pattern was seen in slides stained by two of the labs (DM, AGC/FL). The submucosa lacy-granular pattern was seen only in slides stained by one lab (JK). The epithelial cell nuclear staining pattern was seen in slides stained by two labs (TB, AGC/FL).

### Interobserver reliability and intraclass correlation coefficient

Interobserver reliability was generally moderately acceptable except for some staining morphology types in two laboratories (TB and AGC/FL, Table 3). Judge’s results were excluded when submucosa was not present, for those staining morphologies dependent on the presence of submucosa (DM-PV, JK, AGC/FL-PV). Submucosa was not present in 6 out of 48 slides.

**Table 3:** Intraclass correlation coefficient (ICC) for judge ratings of staining densities, for each testing laboratory (DM, TB, JK) and the central laboratory (AGC/FL), of each staining morphology type (LP = lamina propria granular pattern; EC = epithelial cell nuclear pattern; LG = lacy-granular pattern; PV = perivascular-vascular pattern)

	Box 1 DM-LP	Box 2 TB-EC	Box 2 TB-LP	Box 3 JK-LG	Box 3 JK-LG^a^	Box 4 AGC/FL-EC	Box 4 AGC/FL-LP	Box 4 AGC/FL-PV
*n*	12	12	12	12	11	12	12	12
Four Raters								
ICC	0.73	−0.04	0.02	0.81	0.78	NA	0.53	NA
Three Raters								
ICC	0.88	−0.05	0.00	0.79	0.72	0.16	0.78	0.80

Some judges did not rate some staining patterns for some laboratories; results are therefore given for four judges where all judge scores were available and for the three common judges when one or more judge scores were missing. Cases without submucosa in the slide were excluded from results for staining morphologies dependent on the presence of submucosa (Box 3, JK-LG)

^a^Excluding insufficient submucosa

Intraclass correlation coefficient for judge ratings of staining densities, for each laboratory and staining morphology type is shown in Table 3. Some judges did not rate some staining patterns for some laboratories. Results are therefore given for four judges where all judge scores were available and for the three common judges when one or more judge scores were missing. Cases without submucosa in the slide were excluded from results for staining morphologies dependent on the presence of submucosa (DM-PV, AGC/FL-PV, JK-LG).

Diagnostic accuracy for identifying PD cases, of judge ratings of staining densities, for each laboratory and staining morphology type were calculated (Table 4). Cases without submucosa in the slide were excluded from results for staining morphologies. Sensitivity were often acceptable (>80 %) but specificity too weak. None of the tested method or staining pattern had a specificity and sensitivity more than 80 % regarding to PD.

**Table 4:** Diagnostic accuracy for identifying PD cases, of judge ratings of staining densities, for each laboratory (DM, TB, JK, AGC/FL) and staining morphology type (LP = *lamina propria* granular pattern; EC = endothelial cell nuclear pattern; LG = lacy-granular pattern; PV = perivascular-vascular pattern). Results for the average of judge’s ratings as well as the “best” judge’s ratings are shown. Some judges did not rate some staining patterns for some laboratories; results are therefore given for four judges where all judge scores were available and for the three common judges when one or more judge scores were missing. Cases without submucosa in the slide were excluded from results for staining morphologies dependent on the presence of submucosa (DM-PV, AGC/FL-PV, JK-LG). AUC = area under curve

	Box 1 DM-LP	Box 1 DM-LP^a^	Box 2 TB-EC	Box 2 TB-LP	Box 3 JK-LG	Box 3 JK-LG^a^	Box 4 AGC/FL-EC	Box 4 AGC/FL-LP	Box 4 AGC/FL-LG
*N*	12	7	12	12	12	11	12	12	12
Four Raters									
AUC	0.63	0.65	NA	NA	0.58	0.53	NA	0.67	NA
Sensitivity	86 %	83 %	28 %	28 %	89 %	97 %	NA	81 %	NA
Specificity	25 %	0 %	75 %	83 %	17 %	17 %	NA	33 %	NA
Youden Index	0.11	−0.17	0.03	0.11	0.06	0.14	NA	0.14	NA
Three Raters									
AUC	0.62	0.53	NA	NA	0.58	0.51	0.71	0.67	0.69
Sensitivity	85 %	78 %	37 %	15 %	89 %	100 %	74 %	74 %	70 %
Specificity	33 %	0 %	67 %	100 %	11 %	11 %	56 %	22 %	44 %
Youden Index	0.19	−0.22	0.04	0.15	0.00	0.11	0.30	−0.04	0.15
Best Rater									
Rater	DM	JK	JL	JL	JK	DM	JL	JK	DM
AUC	0.61	1.00	0.50	0.72	0.59	0.46	0.83	0.67	0.80
Sensitivity	100 %	100 %	78 %	44 %	89 %	100 %	67 %	100 %	78 %
Specificity	33 %	0 %	33 %	100 %	33 %	33 %	100 %	67 %	67 %
Youden Index	0.33	0.00	0.11	0.44	0.22	0.33	0.67	0.67	0.44

^a^Excluding insufficient submucosa

## Discussion

The primary aim of this study was to evaluate and compare different IHC methods for the detection of LTS in colonic biopsies. Regarding the ability to discriminate between control and PD subjects, the overall impression is that this was not accomplished by any of the staining methods or morphologically-defined staining types.

One of the striking results of our study is that positive alpha-synuclein staining was observed by all 4 judges in most of the slides from control cases, regardless of the staining methods that were used. Although several studies showed a high sensitivity of FFPE gastrointestinal biopsies for the detection of LTS in PD patients [13, 15], recent reports have raised concerns regarding the specificity of this approach because alpha-synuclein immunoreactivity was also observed in some healthy individuals without PD [17, 20]. This lack of specificity may result from technical difficulties inherent in the use of FFPE gastrointestinal biopsies for the detection of alpha-synuclein. Besides neurons, alpha-synuclein is physiologically expressed by red blood cells and vascular endothelial cells [21, 22], two cell types, which can be retrieved from a routine gastrointestinal biopsy and thus be detected by alpha-synuclein immunohistochemistry in both healthy subjects and PD patients. Moreover, the interpretation of alpha-synuclein immunostaining for the detection of enteric LTS in PD can be delicate because of the scarcity and the morphology of the lesions. Rare typical Lewy bodies have been reported in the outermost plexus of the gut, namely the myenteric plexus, which can not be reached with a routine gastrointestinal biopsy [8, 17, 20]. By contrast, the mucosa and the submucosa, which are captured by the biopsy-forceps, primarily contain thread-like and dot-like alpha-synuclein deposits in nerve endings, reminiscent of the small-sized Lewy neurites observed in the brain [8, 12, 23, 24]. Such a morphology of staining can make it difficult to differentiate between LTS and physiological and/or non-specific alpha-synuclein staining in endoscopy tissue samples.

The above-mentioned limitations outline the critical need to develop proper neuronal counterstaining for the analysis of LTS in gastrointestinal biopsies. Combining routine colonic biopsies and microdissection techniques, we have previously shown that collecting whole-mounts of submucosa and mucosa enable a comprehensive assessment of the neural networks of these two structures [11, 12]. In such whole mount-preparations, immunohistochemical analysis with a general neuronal marker such as PGP9.5, NF220 and Hu revealed that a single standard colonic biopsy contains an average of 150 submucosal neurons and a dense network of nerve fibers in the mucosa [11, 24]. Although this approach provides outstanding information on the morphology of enteric neurons and allows the detection of Lewy pathology in most PD subjects, it nonetheless has several limitations as it has to be performed quickly following the endoscopic procedure, requires an experienced technician and is not suitable for retrospective analysis. It is therefore unlikely that such a procedure will be used in large-scale multicenter studies and there is a clearly need for reliable ways to label neuronal structures in FFPE gastrointestinal biopsies. Most of the available studies performed on FFPE blocks did not use any neuronal markers and relied primarily on morphology to identify the neuronal processes [13, 16, 17]. Only two studies used S100 staining to exclude a significant proportion of biopsies from analysis due to the lack or paucity of nerve fibers [14, 15]. For specificity reason, we would however not recommend the use of S100 staining for the detection of the neuronal processes as this protein is widely expressed by enteric glial cells, which are found in routine gastrointestinal biopsies [25]. Our preliminary observation suggests that immunostaining with the pan neuronal marker PGP9.5 is an efficient screening method to establish whether the tissue sample contains an adequate number of neuronal structures.

In the current study, 3 out of 4 participating laboratories used antibodies against alpha-synuclein phosphorylated at serine 129 to detect LTS. Since the discovery by Fujiwara and coworkers that this residue was selectively and extensively phosphorylated in synucleinopathy lesions [26], antibodies for phosphorylated alpha-synuclein have been widely used to label LTS in the central nervous system as well as in peripheral autonomic networks including the enteric nervous system [3, 9]. Regarding gastrointestinal biopsies, most of the existing studies have used phospho-alpha-synuclein rather than total alpha-synuclein antibodies for the detection of pathological alpha-synuclein. Although some studies reported that staining with phospho-alpha-synuclein antibodies to be more extensive to that obtained with total alpha-synuclein [14, 27], other studies found the opposite [15, 16]. This may be due to the fact that a small amount (around 4 %) of alpha-synuclein is physiologically phosphorylated at serine 129 [26], implying that phospho-synuclein antibodies can detect physiologic non-aggregated protein when no pretreatment is used. In this context, Visanji et al. recently adapted the paraffin-embedded tissue (PET) blot method, which degrades physiologic nonaggregated proteins and has been demonstrated to have superior sensitivity in detecting aggregated protein, to the detection of LTS in gastrointestinal samples [16]. Surprisingly, they observed phospho-alpha-synuclein immunostaining in the colonic mucosa of 11/11 of control subject, despite the use of the PET blot method. This strongly suggests that non specific staining occurs in the gastrointestinal mucosa when phospho-alpha-synuclein antibodies are used, thereby explaining the lack of specificity observed in our study.

One obvious limitation of our study is the small sample size of the control group and it is possible that if it was repeated with more controls, the diagnostic accuracy of some of the methods might be higher. Another limitation was the absence of submucosa, blood vessels or judge’s scores for most of the slides, and for two of three control cases, rated for the perivascular staining morphology produced by the one testing laboratory (DM). It is possible, if all three controls had been graded and the staining scores segregated with the appropriate diagnosis, that the results for this laboratory may have been in fact acceptably accurate. Arguing against this possibility, however, is the low accuracy for this same morphology as produced by the central laboratory.

## Conclusions

In conclusion, although our study has produced results that are less than definitive but it suggests that the tested immunohistochemical methods are not adequate for the prediction of PD in endoscopically obtained gastroinestinal biopsies. This failure may be due to the relative rarity of neuronal synucleinopathy in the submucosa of the colon or to the lack of sensitivity and specificity of the methods tested. Future studies including more control subjects along with new staining methods and screening for neuronal markers are needed.

## Additional file

Additional file 1: Figure S1. Templates used for semi-quantitative grading of staining morphologies present in the test sections. **Figure S2.** Representative Hematoxylin and Eosin staining of a colonic biopsy defining the regions available for assessment (mucosa and submucosa). Scale bar 200 μM. **Table S1.** Results of individual judge scores for the staining done by each test laboratory as well as by the central laboratory, for each scoring template. (PDF 3317 kb)
