# Supplementary material for: Evaluation of alpha-synuclein immunohistochemical methods for the detection of Lewy-type synucleinopathy in gastrointestinal biopsies
Source: Acta Neuropathol Commun. 2016 Apr 4;4:35. doi: 10.1186/s40478-016-0305-8 (PMC4820972; doi:10.1186/s40478-016-0305-8)
Supplement: Additional file 1: Figure S1. — Templates used for semi-quantitative grading of staining morphologies present in the test sections. Figure S2. Representative Hematoxylin and Eosin staining of a colonic biopsy defining the regions available for assessment (mucosa and submucosa). Scale bar 200 μM. Table S1. Results of individual judge scores for the staining done by each test laboratory as well as by the central laboratory, for each scoring template. (PDF 3317 kb) [file 40478_2016_305_MOESM1_ESM.pdf]

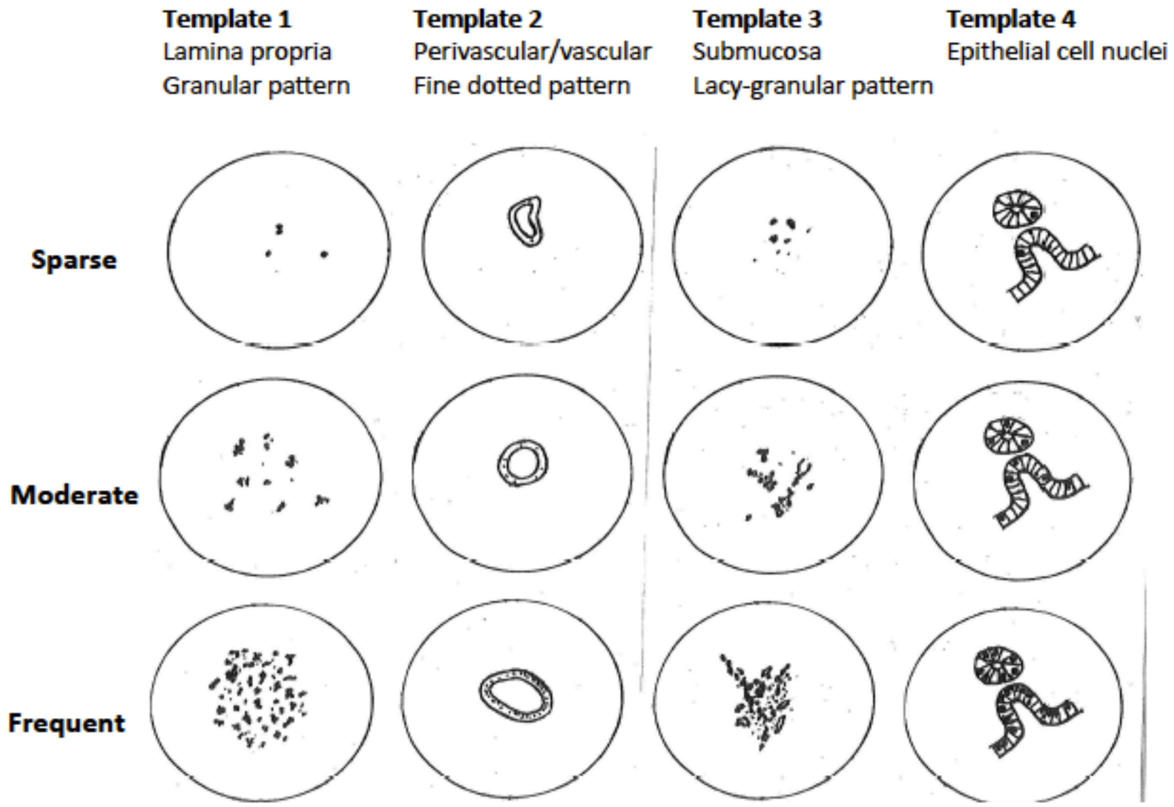

Supplementary Figure 1. Templates used for semi-quantitative grading of staining morphologies present in the test sections.

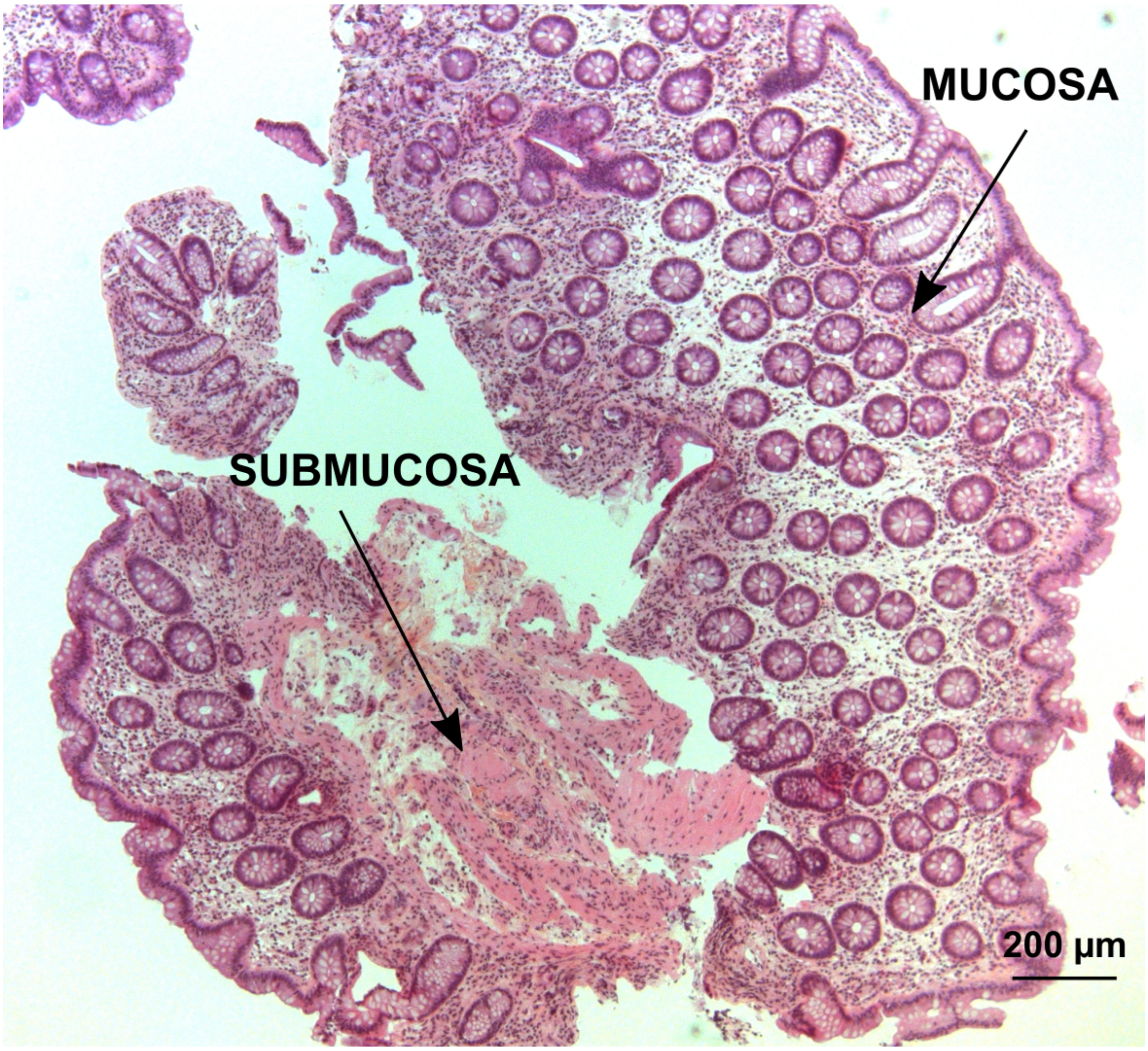

Supplementary Figure 2. Representative Hematoxylin and Eosin staining of a colonic biopsy defining the regions available for assessment (mucosa and submucosa). Scale bar 200 µM.

TEMPLATE 1			
	TB	DM	AGC
Control 1	0,1	0,1	0,2
Control 2	0,1	2,2	1,4
Control 3	0,0	0,9	0,4
controls (mean)	***0,1***	***1,0***	***0,7***
PD 1	0,1	0,4	0,3
PD 2	0,1	1,3	0,4
PD 3	0,0	0,5	0,2
PD group 0 (mean)	***0,0***	***0,7***	***0,3***
PD 4	0,1	1,8	1,4
PD 5	0,2	0,8	0,9
PD 6	0,1	1,5	1,7
PD group 1 (mean)	***0,1***	***1,3***	***1,3***
PD 7	0,2	2,8	1,7
PD 8	0,1	2,1	1,8
PD 9	0,0	2,8	1,6
PD group 2 (mean)	***0,1***	***2,6***	***1,7***

TEMPLATE 2	
	AGC
Control 1	0,5
Control 2	0,6
Control 3	0,3
controls (mean)	***0,5***
PD 1	0,0
PD 2	2,7
PD 3	2,1
PD group 0 (mean)	***1,6***
PD 4	1,9
PD 5	2,4
PD 6	0,6
PD group 1 (mean)	***1,6***
PD 7	0,0
PD 8	0,8
PD 9	0,2
PD group 2 (mean)	***0,3***

TEMPLATE 3	
	JK
Control 1	0,3
Control 2	3,0
Control 3	2,3
controls (mean)	***1,9***
PD 1	0,1
PD 2	1,4
PD 3	1,1
PD group 0 (mean)	***0,9***
PD 4	3,0
PD 5	2,0
PD 6	2,4
PD group 1 (mean)	***2,5***
PD 7	0,5
PD 8	2,0
PD 9	2,5
PD group 2 (mean)	***1,7***

TEMPLATE 4		
	DM	AGC
Control 1	0,0	0,4
Control 2	0,4	0,8
Control 3	0,3	0,8
controls (mean)	***0,2***	***0,7***
PD 1	0,5	2,3
PD 2	0,6	0,7
PD 3	0,4	1,1
PD group 0 (mean)	***0,5***	***1,3***
PD 4	0,1	1,2
PD 5	0,3	1,0
PD 6	0,2	1,6
PD group 1 (mean)	***0,2***	***1,3***
PD 7	0,0	0,4
PD 8	0,2	1,1
PD 9	0,2	1,0
PD group 2 (mean)	***0,1***	***0,8***

Supplementary Table 1. Results of individual judge scores for the staining done by each test laboratory as well as by the central laboratory, for each scoring template.
